# Hepatitis C in Israeli prisons: status report

**DOI:** 10.1186/s12954-020-00430-y

**Published:** 2020-10-19

**Authors:** Niv Michaeli, Anat Litvin, Nadav Davidovitch

**Affiliations:** 1 Physicians for Human Rights Israel, Tel Aviv, Israel; 2 School of Public Health, Faculty of Health Sciences, Ben Gurion University of the Negev, Beersheba, Israel

**Keywords:** HCV, Prison, Israel, Human rights

## Abstract

The article discusses and analyzes the changes that have taken place since 2016 in Israeli policy with regard to the treatment, diagnosis and detection of hepatitis C (HCV) in prison settings. The article finds indications of promising changes to official procedure, such as the statement by authorities that they plan to begin screening new inmates for HCV, and the increase in the number of inmates provided antiviral drugs. These measures, however, only came about after a prolonged campaign and legal battle by human rights organizations, patient advocacy groups and the medical community. Despite these encouraging changes, it appears that a significant portion of inmates in need of treatment are still not getting it due both to bureaucratic delays and to inmates’ reluctance. In addition, in the absence of a suitable screening program, the extant figures of morbidity—high in themselves—may reflect underdiagnosis. The flaws in the policymaking process and in its implementation may be attributed, at least in part, to the fact that the prison healthcare system is under the aegis of the Israel Prison Service and not that of a medical body. This reality places the medical staff in prisons in a state of dual loyalty, and also means the prison healthcare system is excluded from national health plans and major sources of budgeting, leaving it without sufficient means to provide the necessary level of care. These problems plague the prison healthcare system in general and are not limited to its handling of HCV. These challenges are not unique to Israel, and many other Western countries must also face the obstacles that are the result of prison healthcare services being subject to the authority of the correctional establishment. As this test case demonstrates, extended active involvement by civil organizations and the medical community are essential to promoting and ensuring inmates’ right to health.

## Forward

A great deal of medical research has shown a higher incidence of hepatitis C (HCV) among prison inmates. Consequently, many health organizations—including the World Health Organization (WHO) [1], the European Centre for Disease Prevention and Control [2], the American Association for the Study of Liver Diseases and the Infectious Diseases Society of America [3], consider prison inmates to be at a heightened risk for HCV. They therefore recommend a proactive policy to detect the virus among inmates. Despite these recommendations and despite Israel’s undertaking in joining the WHO initiative to eliminate the virus by 2030, Israel has thus far refrained from establishing a clear policy regarding inmates. Moreover, in spite of substantial steps towards making antiviral drugs available in the community, the portion of inmates receiving these medications remains low and they face many obstacles in obtaining treatment. This paper will describe IPS policy regarding screening for and treating HCV and will also discuss promising indications that—after years of campaigning—changes to this policy are finally beginning to take shape. In addition, the present paper will illustrate the injurious impact of the prison healthcare system being administered by the security establishment. Finally, it will discuss the crucial role that the medical community and civil society play in promoting inmates’ right to health.

## Israel’s prison healthcare system

The Israel Prison Service (IPS), the national body responsible for Israel’s prisons, operates 31 incarceration facilities. In April 2020, there were some 14,000 inmates in IPS facilities, including approximately 4,500 Palestinians whom Israel has classified as “security prisoners.” Israel’s National Health Insurance Law (1994)—which stipulates that all Israeli residents are entitled to a package of publically funded health services—does not apply to inmates. Instead, they are entitled to medical care by the IPS, as set out in the Prison Ordinance.

In each of its incarceration facilities, the IPS operates a medical clinic which is staffed by a doctor and a team of several EMTs (emergency medical technicians). As the doctors and the other medical personnel are employed directly by the IPS, they are placed in a conflict of dual loyalty. Although prison physicians fulfill the role of primary care providers, like family doctors in the community in Israel’s HMOs (Hebrew: *kupot holim*), IPS physicians are general practitioners not certified in any medical specialty. This is a far cry from the situation in Israel’s public health system in which over half of family physicians are specialists. This difference is significant and problematic, particularly in view of the high rate of morbidity among inmates in Israel. As in many other countries [4], also in Israel the rate of morbidity among inmates is higher than in the general population. According to IPS figures, some 40% of inmates have been diagnosed with at least one chronic illness^1^ and some two-thirds of criminal inmates have a history of alcohol or drug addiction [5]. This medical situation increases the IPS’s need to refer inmates to external specialists, either by taking the inmates to hospitals or by having specialists make visits to the prisons. As a result of limitations on the availability of specialists and the difficulty in conveying inmates to hospitals—either for security reasons or due to staffing constraints—inmates’ wait times for examination and treatment can be as much as seven to twenty times as long as in the community,^2^ and could jeopardize treatment continuity.

Excluding inmates from the National Health Insurance Law has two major consequences. First, the Ministry of Health has no oversight authority of the prisons nor can it prescribe policy. Second, whereas the general public in Israel has a right to a predetermined medical services package whose parameters are known and established in law, the Prison Ordinance and internal IPS directives do not stipulate a fixed medical package, leaving matters mostly to the discretion of the IPS. Over the years, court rulings on inmate petitions that appealed IPS decisions denying the inmates various treatments have stated that the IPS is obliged to provide inmates treatment in accordance with the medical service package provided in the community.^3^ This is a very important issue as the medical services package provided under the National Health Insurance Law is updated annually, and the HMOs are given specific budgeting to cover new treatments added to the package. The IPS, however, while it is required to match the package, it is not included in the law’s budgeting mechanism.

## Israel’s policy for screening, treatment and diagnosis of hepatitis C

As of now, no methodical HCV screening procedure has been put in place in the community. Studies have shown a prevalence of 1.2% [6] to 2% [7] of HCV in the general population. Research has also found a higher incidence of HCV among immigrants from the FSU (Former Soviet Union) than in the overall population in Israel. This fact is important because of their over-representation in prisons in Israel.

In 2016, the Ministry of Health stated that Israel is taking part in the WHO initiative to eliminate HCV by 2030 and would be promoting a plan for carrying out testing among groups who are at higher risk of contracting the virus [8]. The press release they issued listed three vulnerable groups: recipients of blood transfusions prior to 1992 (when the blood bank began screening donated blood for the virus); immigrants from the former USSR; and individuals who have engaged in intravenous drug use. Although four years have gone by since this declaration by the Ministry of Health, a clearly structured national program for detecting viral hepatitis in the risk groups has yet to be implemented in Israel. At the same time, there may be some basis for cautious optimism that such a plan will be launched in the near future. In November 2019 the Ministry of Health issued a draft of a brief regarding the detection and treatment of individuals with HCV, instructing the HMOs to offer testing to all patients in risk groups. In addition to the groups listed in its 2016 statement, the draft brief added former inmates as a risk group.

While progress has been extremely limited and slow in terms of testing policy, HCV-positive individuals’ access to DAAs treatment has greatly expanded in recent years. As of January 2018, there is full public coverage for treatment regardless of the genotype or severity of fibrosis. As of 2015 some of the medications have begun to be included in the public medical services package, first to patients with advanced fibrosis and incrementally expanding to cover all HCV carriers.

## Availability of hepatitis C testing in the Israel prison service

Although the IPS is required to provide inmates with the same treatment that the Israeli public healthcare system normally provides, many inmates in need of treatment have faced great obstacles in obtaining it. The issue of providing the new therapy to inmates first arose in late 2015 when a criminal inmate diagnosed with advanced fibrosis (F3) appealed to the district court, seeking to require the IPS to provide him with the treatment recommended by the hepatologist he had been referred to by the IPS.^4^ Initially, the IPS took the position that it should not be required to provide the treatment since the IPS was not allocated a budget to cover this therapy. Later, the IPS later also argued that the inmate would not be in an imminent life-threatening situation if he does not receive treatment. The court rejected the IPS position and established that the IPS is required to provide inmates with treatment according to the medical package available in the community to the general public. After this decision, while the IPS has refrained from openly declaring that it was not providing treatment for HCV, it has continued to impose significant obstacles to inmates in need of treatment. Inmates who were diagnosed as carriers were not sent to complete the battery of tests and assessments essential to initiating treatment, or else the examination process was protracted and unduly delayed. A case in point is one in which Physicians for Human Rights (PHRI) represented an inmate in an appeal to the district court: As part of the inmate’s assessment in a liver clinic, he had undergone a FibroTest that found he had advanced fibrosis (F3–F4). Nevertheless, ten months passed from the test until he started receiving the necessary medicine because of delays in getting him to the liver clinic to bring the test results and in completing some other basic tests, such as an abdominal ultrasound.^5^ In 2019, PHRI represented another inmate in a petition to the district court, which sought that he be provided the treatment the liver clinic recommended six months earlier.^6^

According to IPS figures, in January 2019 there were 435 HCV-positive inmates.^7^ The following breakdown was given: PCR testing determined that 105 inmates did not require drug therapy; 53 had completed drug therapy; 76 were listed as having refused further testing or refused starting treatment despite testing positive for antibodies; and 201—nearly half of the inmates found to be HCV-positive—were listed as in the process of assessment for beginning treatment. The information the IPS conveyed did not state what examinations these inmates were waiting for.

Although on January 1, 2019, the IPS classified 201 inmates as being in the process of examination in preparation for beginning drug therapy, according to figures the IPS published, only 53 inmates received treatment in 2019.^8^ In other words, most of the inmates labeled as being in the process of completing examinations, had not completed the process, either because of the pace at which matters were being handled by the IPS, or because the inmates were not interested in pursuing the process, or because they had not yet been provided the drug therapy despite having completed the necessary assessment.

Contracts between the IPS and pharmaceutical companies indicate that the IPS has allocated a sum that would cover the treatment of approximately 100 inmates in 2020; this is in addition to inmates during their first year of incarceration whose treatment is financed by their HMOs. The costliness of the medications (58–63,000 Israeli shekels, equivalent to approximately 17–18,000 USD per inmate) poses a considerable challenge. Under these contracts, the pharmaceutical companies gave the IPS discounts on the medications. The contracts also included a provision that should the IPS provide treatment to more than 160 inmates over the course of two years, the company would finance treatment for another 40 inmates. Although such cooperation between the state and pharmaceutical companies is clearly an important issue, this paper will not elaborate on it. Nevertheless, we do wish to emphasize that this collaboration gives rise to dilemmas with regard to devising strategies for testing and treatment [9] and more broadly to dilemmas regarding the responsibility of the state to guarantee medical services.^9^

## IPS screening policy for hepatitis C

The above-mentioned IPS figures—435 inmates who had tested positive for HCV antibodies—represent about 5% of criminal inmates in Israeli prisons. This number, despite being high in and of itself—more than double of that in the community—may yet reflect a state of under-diagnosis. As of June 2020, it is known that the IPS does not carry out proactive testing to detect the virus in the inmate population. The IPS carries out tests only when there are medical indications of liver disease or when inmates are referred for surgical procedures.

As early as 2016—after the Ministry of Health published its statement regarding advancing a national program for screening at-risk populations—PHRI, together with several other organizations which promote patient rights, jointly applied to the Ministry of Health, demanding that it takes action to incorporate the IPS in the initiative. The Ministry of Health responded by stating that it has no authority over health policymaking in the prisons. Yet similar applications to the IPS were met with the response that the IPS was not undertaking screening tests among the inmates because it had received no such instructions from the Ministry of Health and that the ministry had not classified inmates as an at-risk group.

In 2018, PHRI petitioned the High Court of Justice, demanding that the Ministry of Health and the IPS be compelled to carry out testing to detect the virus among inmates. The authorities’ position underwent several changes in the course of the hearings in the petition. In its initial response, filed in March 2019, the state argued that it opposes a sweeping classification of inmates as an at-risk population, and that there were no indications of instances of contagion in the prisons. The state added that it is slated to begin running a “smart algorithm” in the HMOs in the next few months. The algorithm is designed to help identify which patients are part of at-risk groups, and that after a pilot period the state will consider implementing the program in the IPS as well. In September 2019, the state informed the Court that due to budgetary issues and technological difficulties, there is currently no estimated start date for beginning the implementation of the smart algorithm, but that in view of the efforts being made by the authorities, the petitions should be denied.

The Court criticized the authorities for foot-dragging. Consequently, in January 2020, the state said that it is taking action to establish a procedure for conducting screening tests among inmates according to the principles set out by the Ministry of Health in November 2019. Despite these declarations, the testing policy instituted by the IPS was markedly different in a number of matters. Whereas the draft of the brief sent out by the Ministry of Health instructed the HMOs to test all former convicts, IPS policy stated that testing be offered only to new prisoners upon arrival and only if they belong to one of the at-risk groups, i.e., intravenous drug users, immigrants from the former USSR or individuals who received blood transfusions prior to 1992. As the response of the state shows, the rationale underpinning the discrepancy in the criteria is financial, rather than medical. The state emphasized that since the testing would be of new inmates, the responsibility for footing the bill for the screening test, and if necessary for further examinations and drug therapy during the first year of incarceration, would be that of the HMO in which the prisoner was a member before imprisonment.

Later, after PHRI submitted to the Court its reservations over the proposed plan, the state announced in June 2020 that it plans to begin running tests to detect carriers among new inmates as of August 2020, and later expand the availability of testing to the entire prison body. In its response, the state wrote that as of March 2021, it plans to put up signs in the prison clinics that would provide explanations about the disease and inform inmates of their right to request to be tested. Notwithstanding this statement, the state reiterated its position that in the absence of evidence of contagion in prisons, inmates should not be considered an at-risk group per se. In view of the change in state policy and the progress made in the matter, the Court dismissed the petition.

## The role of the medical community in changing policy

As in many other countries, there is a clear separation in Israel between the bodies responsible for medical services for inmates and those responsible for healthcare in the community. One clear sign of this division is the scant attention paid by the medical community in Israel to the situation in the prisons. It is a matter that is usually not addressed in national healthcare policymaking or in research by healthcare personnel. We believe that one of the reasons for the success—albeit partial—of the struggle to promote the rights of HCV-positive inmates in Israel, along with the change in the authorities’ policy, is the involvement of various officials and members of the medical community.

In recent years, in addition to the legal battle in the courts, the issue of HCV in prisons has received regular and constant attention by civil society organizations. They helped initiate Knesset (Israel’s parliament) committee sessions devoted to the matter and also media coverage. In addition to PHRI, which has over 2000 members from the medial professions, other patient-oriented organizations—such as the NGOs Hetz (for the rights of liver patients) and the Israel AIDS Task Force—proactively promoted the issue.

In October 2018, the National Council for Gastroenterology, Hepatology and Nutrition—a committee of experts which advises the Ministry of Health—issued a recommendation to conduct testing to detect HCV among inmates. A number of doctors who are members of both the National Council and the Israel Association for the Study of the Liver have often taken part in the Knesset sessions and highlighted the importance of this issue. The Israel Association for Public Health Physicians of the Israeli Medical Association was similarly very involved. In 2016, it called on the IPS to provide treatment to inmates, and in January 2020 published a position paper on this subject which was submitted to the High Court of Justice as part of the petition to oblige the state to carry out screening tests [10]. In its paper, the Israel Association for Public Health Physicians voiced its support for the demand to conduct universal testing among inmates. The paper added that despite the concern regarding under-diagnosis among the inmates because of refraining from testing, the extant figures already show an increased prevalence of the disease among inmates and this justifies classifying them as a risk group. They also felt that the high number of inmates who refuse testing or treatment and the high number of inmates in assessment process reflect shortcomings in educating inmates about HCV and making information accessible to them, and raise concerns regarding unnecessary foot-dragging prior to commencing treatment.

## Conclusions

After years that civil society organizations and members of the medical community have waged this struggle, we can now see indications of positive trends in testing policy in Israeli prisons and an increase (although still inadequate) in the number of inmates receiving treatment. We must still wait for the implementation of the new testing policy and find out the extent of inmates’ cooperation before we can know to what extent HCV education in the prisons has been successful.

Alongside cautious optimism, an examination of Israeli authorities’ policy vis-à-vis hepatitis C in prisons leads to several insights that are more broadly relevant to healthcare policy in prisons. The fact that prison doctors are employed directly by the IPS and are subject to the authority of the security establishment places them in a state of dual loyalty. This conflict of loyalties can be seen when doctors make decisions that are at odds with customary professional standards, instead aligning with the financial interests of their employer, the prison system. Organizationally, placing the IPS healthcare system under the Ministry of Public Security rather than under the Ministry of Health has resulted in the latter not having clear authority to dictate prison policy. In addition, it also means that significant measures on the national policy level, such as allocating budgets to the healthcare system to cover new drugs, are not applied to the IPS. Finally, although the inmates are a small, focused group with a high prevalence of hepatitis C, Israeli authorities have refrained from taking action in prisons. Instead, they have chosen to defer handling the situation in prisons until after the implementation of a far more complex screening plan in the community, a plan which has been delayed for over four years. The lack of commitment by the authorities to promote prisoners’ health is not unique to its conduct in the case of HCV. Healthcare services to inmates in Israel are currently in a severe crisis. Solving it requires a major change on the part of the Ministry of Health and the IPS [11].

## Data Availability

The datasets used and/or analyzed during the current study are available from the corresponding author on reasonable request.

## Footnotes

1 Data conveyed from the IPS to PHRI, dated January 1, 2019, under a Freedom of Information Request.

2 In an interview for the IPS magazine in 2010, with then IPS Chief Medical Officer Dr. Dini Tishler-Aurkin.

3 Prisoner Petition 2808/05, *Tamimi v. Director of Medical Department*.

4 Prisoner Petition 28,629–08-15, *Rosalio v. IPS.*

5 Prisoner Petition 21,852–04-18.

6 Prisoner Petition 67,259–01-19.

7 According to IPS figures conveyed to PHRI on January 1, 2019 in response to a Freedom of Information Request.

8 The numbers were taken from the contract between IPS and Teva Pharmaceutical Industries, signed January 7, 2020.

9 Nadav Davidovitch, “Public–Private Partnerships as a Strategy FOR Migrant Inclusion – Stopgap or Foot in the Door?” Commentary on: Ensuring HIV Care to Undocumented Migrants in Israel: A Public–Private Partnership Case Study (forthcoming).
